# Rapid-Onset Obesity, Hypothalamic Dysregulation, Hypoventilation, Autonomic Dysregulation, and Neuroendocrine Tumour: Rare Syndrome with Myriad Anaesthesia Implications

**DOI:** 10.5152/TJAR.2022.21395

**Published:** 2022-12-01

**Authors:** Priyanka P. Karnik, Nandini M. Dave, Majid Sayyed, Vaibhav Dhabe

**Affiliations:** 1 Department of Anaesthesiology, NH-SRCC Children’s Hospital, Keshavrao Khadye Marg, Mahalakshmi, Mumbai, India

**Keywords:** Airway management, depth of anaesthesia, obesity, paediatric anaesthesia, ROHHAD syndrome

## Abstract

Anaesthesia management of a child with rapid-onset obesity, hyperphagia, and hypothalamic dysfunction syndrome is complex due to the multisystem involvement, the most important features being morbid obesity, autonomic dysfunction, and dyselectrolytemia due to hypothalamic dysfunction. The acronym of the disease was amended in 2008 to rapid-onset obesity, hyperphagia, hypothalamic dysfunction neural crest tumour to include the risk of ganglioneuroma or ganglioneuroblastoma. Patients usually require removal of tumour in the prone position. Obstructive sleep apnea, difficult airway and intravenous access, and haemodynamic lability all add to the trials faced by the paediatric anaesthesiologist. Invasive haemodynamic monitoring, ultrasonography, bispectral index monitoring, and meticulous calculation of drug dosages help in smoothening the course of anaesthesia in the presence of constant vigilance.

**Main Points**

- The management of these patients requires a multidisciplinary approach.
- The paediatric anaesthesiologist is faced with the challenges of difficult airway, vascular access, positioning, and maintenance of stable hemodynamics during the intraoperative period.
- Further reports of such cases are needed for formulating management guidelines.

## Introduction

A 3-year-old female child weighing 40 kg (height of 100 cm and body mass index [BMI] of 40 kg m^-2^), a suspected case of rapid-onset obesity, hypothalamic dysregulation, hypoventilation, autonomic dysregulation, neuroendocrine tumour (ROHHADNET) syndrome was posted for tumour excision. Rapid severe weight gain due to hypothalamic dysfunction is the harbinger of the disease followed by signs of autonomic dysfunction, hypoventilation, and behavioural symptoms. Although there is literature mentioning various aspects of ROHHAD syndrome, we found only a couple of case reports mentioning anaesthesia implications.^1,2^ Written informed consent was taken from the patient’s parent for this case report.

### Case Presentation

The patient was apparently healthy till 2 years of age after which she started gaining weight rapidly. The weight gain was as much as 29 kg in 1 year, about 10 kg in 3 months. She presented with hyperphagia and symptoms of autonomic dysregulation like hypertension, baseline tachycardia, constipation, and temperature dysregulation. She also presented with uncontrolled diabetes mellitus requiring oral hypoglycaemic drugs and later insulin therapy. She also had frequent urinary tract infections. She had a history of snoring and night-time awakenings. Her polysomnographic examination showed severe obstructive sleep apnoea (OSA) with Apnoea Hypopnoea Index of 12.8 and night-time denaturation up to 58%. Echocardiographic examination showed mild pulmonary hypertension. Congenital central hypoventilation syndrome (CCHS) was ruled out as she tested negative for the POHX2B gene. Prader–Willi syndrome, Cushing’s syndrome, and Beckwith-Weidemann syndrome had also been ruled out. The patient was referred to an endocrinologist in order to optimise the blood sugar levels prior to surgery. Adrenocorticotropic hormone value was within the normal limits. Computed tomography of the abdomen pelvis reported a well-defined tumour in the sacral region measuring 5 × 3.5 × 5 cm.

**Figure 1.**
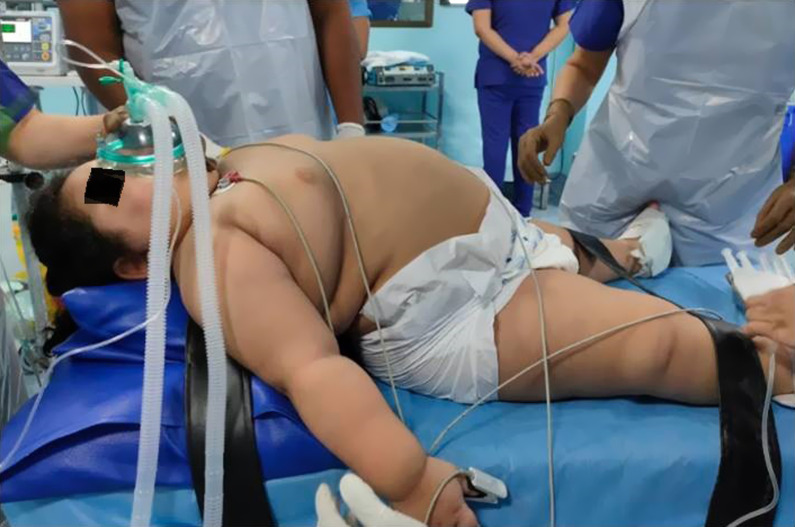
Ramped position at induction of anaesthesia.

The patient’s adjusted body weight (23 kg) and ideal body weight (15 kg) were calculated from the total body weight using appropriate formulae for drug dosage calculations. The operating room (OR) was equipped with a difficult airway trolley due to the possibility of difficult mask ventilation and ultrasonography (USG) machine due to difficult intravenous (IV) access. As the patient had an IV line in situ, she was premedicated with glycopyrrolate 4 μg kg^-1^, midazolam 1 mg, and ketamine 20 mg intravenously. The child was kept on the OR table in the ramped position (Figure 1). The patient was induced with gradually increasing sevoflurane, mask ventilation was confirmed, and a check laryngoscopy was done using Macintosh III curved blade after administering IV propofol 50 mg. Intravenous atracurium 15 mg was given after Cormack Lehane grade II laryngoscopic view was confirmed. The patient was intubated using a 4.5-mm ID microcuff endotracheal tube. Airway pressures were high in view of decreased lung and chest wall compliance. Lung protective ventilation using a pressure regulated volume controlled mode was utilised. Due to difficult IV access, a 5.5 Fr central venous line was inserted under the USG guidance and an arterial line was inserted for beat-to-beat blood pressure monitoring. The child was given a prone position with attention to pressure point padding. As intraoperative neuromonitoring with somatosensory-evoked potentials and motor-evoked potentials was planned, anaesthesia was maintained with a mixture of oxygen, air, and inhalational sevoflurane at less than 0.5 MAC along with dexmedetomidine and propofol infusion. Muscle relaxants were not given after the initial induction dose. Bispectral index monitoring was done to ensure adequate depth of anaesthesia. Five percent dextrose normal saline and Ringer’s lactate were used as the maintenance and replacement fluid, respectively along with titrated insulin infusion based on the sliding scale. Blood glucose levels were monitored hourly. Intravenous morphine 1.5 mg was given for analgesia. Core as well as skin temperature were monitored, and a convective warmer with a blanket was used to prevent hypothermia along with fluid warmer. There was transient syndrome of inappropriate antidiuretic hormone-like picture intraoperatively with fall in urine output which responded to IV furosemide. The intraoperative blood loss was 40-50 mL only. The patient remained euthermic and stable haemodynamically without any fluctuations in temperature or blood pressure. The blood sugar level was maintained in the range of 180-200 mg dL^-1^. The child was electively shifted to paediatric intensive care unit on a ventilator in view of long-duration surgery (9 hours) and severe OSA. She was extubated the next day keeping a continuous positive airway pressure mask on standby for non-invasive ventilation. She was discharged 10 days later and was put on night-time bi-level positive airway pressure which improved her sleep pattern.

## Discussion

Rapid-onset obesity, hyperphagia, and hypothalamic dysfunction syndrome was first described by Ize-Ludlow et al.^3^ It is a diagnosis of exclusion and can be distinguished from CCHS, Beckwith-Weidman syndrome, and Prader–Willi syndrome based on genetic evaluations. There is an associated entity known as ROHHADNET with a predisposition towards the development of neuroectodermal tumours (ganglioneuromas and ganglioneuroblastomas).^4^ The onset of the disease is around 2-4 years of age with sudden onset of dramatic weight gain, dysautonomia, and pulmonary complications.^1,2^ It can be life-threatening with death around 10 years of age due to sudden cardiac arrest.^2^ There are various phenotypic features of ROHHAD syndrome affecting respiratory, cardiovascular, and central nervous system with 40% presenting with a neuroectodermal tumour. Autonomic dysregulation may present as ophthalmologic abnormality such as blurred vision, altered pupil response to light, strabismus, altered perception of pain, gastrointestinal dysmotility with chronic constipation or diarrhoea, bradycardia, neurogenic bladder, excessive sweating, thermal dysregulation (hypothermia and hyperthermia), cold hands and feet, livedo reticularis, pseudo-Raynaud’s phenomenon, syncope, urinary incontinence, and dysarthria.^5^ The treatment is based on clinical features and may include hormone replacement, strict fluid and caloric intake regimen, and exercise. Anaesthesia implications are multitudinous which are summarised in Table 1.

Our patient had a BMI of 40 kg m^-2^ and weight more than 99th percentile for age with symptoms and polysomnography findings of severe OSA, difficult venous access, difficult mask ventilation, and symptoms of dysautonomia like hypertension, constipation, resting tachycardia, and diabetes mellitus. In our case, premedication was administered with caution using minimal doses and close watch over respiration due to the heightened sensitivity to opioids and benzodiazepines.^1^ Appropriate-sized airway devices were kept ready keeping in mind the age as well as the weight as weight alone can be misleading for sizing. Ramped position was extremely useful for airway management not only during this procedure but also when the patient came to us subsequently for peripherally inserted central catheter placement under sedation 2 months later. Obtaining invasive lines was challenging and was made easier with the use of USG. Maintaining normothermia, stable haemodynamics, and monitoring blood sugar levels were of utmost importance in this case for a good postoperative outcome and healing. Due to the heavy abdominal pannus in this patient, soft bolsters with extra padding were used to prevent pressure necrosis in the prone position. Drug dosages were calculated meticulously as pharmacokinetics are altered with an increase in the volume of distribution for lipophilic drugs. In our case, lipophilic drugs like opioids and propofol were given according to the total body weight whereas hydrophilic drugs like muscle relaxants were given according to the ideal body weight.^6^ Postoperatively, these children benefit from non-invasive ventilation especially in children with a history of nocturnal desaturations.^6,8,9^ A multidisciplinary approach is required in these patients where paediatrician, endocrinologist, polysomnologist, pulmonologist, cardiologist, anaesthesiologists, and surgeons need to discuss the treatment modalities, potential risks, preoperative optimisation, and perioperative planning. Being a diagnosis of exclusion and a rather rare entity, we need to report such cases to formulate guidelines regarding preoperative management.^1,2^

## Figures and Tables

**Table 1.** Clinical Signs and Anaesthesia Implications in ROHHAD Syndrome

**Clinical Features**	**Anaesthesia Implications**
Disorders, behavioural	Premedication is a must as patients present with emotional lability. Intravenous midazolam and IV ketamine should be used judiciously.
Disordered breathing	Sleep-disordered breathing is a common finding with many presenting with nocturnal hypoxaemia. Early nocturnal ventilation (BiPAP) helps to improve ventilation.^7^
Difficult IV access	Morbidly obese patients may have difficult intravenous access and need for frequent blood draws for a battery of investigations. Ultrasonography guidance is extremely useful not only for central venous access in a patient with a short neck but also for peripheral venous access.
Difficult airway	Mask ventilation may be difficult due to the submental and buccal fat with a short neck and accompanying severe obstructive sleep apnea. Laryngeal mask airway may help as a rescue. Continuous positive airway pressure may be needed in the perioperative period to maintain airway patency.
Dysautonomia	Rapid sequence induction may be required despite adequate fasting if the patient has gastroparesis. Volume status needs to be optimised since these patients can have precipitous changes in blood pressure in response to blood loss or position change, especially in patients with preoperative orthostatic hypotension. Vasopressors and inotropes can elicit variable response due to either resistance or undue sensitivity.
Diabetes mellitus, diabetes insipidus/SIADH	Endocrinologist reference is essential in cases presenting with DM, hyperprolactinemia, hypothyroidism, and adrenal insufficiency due to hypothalamic dysfunction. Electrolyte disturbances due to SIADH or DI should be corrected preoperatively.
Drug dosages	Drug dosages should be calculated meticulously differentiating drugs to be given according to total body weight, ideal body weight, and adjusted body weight to avoid overdosing or underdosing of drugs which can lead to delayed recovery or intraoperative awareness respectively.
Dysfunction	Preoperative 2D echocardiography gives useful information regarding any dysfunction due to dysautonamia or the presence of pulmonary hypertension in patients with severe OSA. Electrocardiography and even Holter monitoring can help in detecting arrhythmias. Pulmonary function testing can be carried out in cooperative older patients. Temperature dysregulation may be present in patients with symptomatic hypothalamic dysfunction

BiPAP, bi-level continuous positive airway pressure; DM, diabetes mellitus; SIADH, syndrome of inappropriate antidiuretic hormone; DI, diabetes insipidus; OSA, obstructive sleep apnea; IV, intravenous.
